# Preliminary Study on Multi-Scale Modeling of Asphalt Materials: Evaluation of Material Behavior through an RVE-Based Approach

**DOI:** 10.3390/ma17205041

**Published:** 2024-10-15

**Authors:** Ahmed Ibrahim Hassanin Mohamed, Oliver Giraldo-Londoño, Baolin Deng, Zhen Chen, Punyaslok Rath, William G. Buttlar

**Affiliations:** Department of Civil and Environmental Engineering, University of Missouri, Columbia, MO 65211, USA; ogiraldo@missouri.edu (O.G.-L.); dengb@missouri.edu (B.D.); chenzh@missouri.edu (Z.C.); rathp@missouri.edu (P.R.); buttlarw@missouri.edu (W.G.B.)

**Keywords:** representative volume element, heterogeneous model, asphalt pavement, fracture, finite element modeling, RVE-based approach

## Abstract

This study employs a microstructure-based finite element modeling approach to understand the mechanical behavior of asphalt mixtures across different length scales. Specifically, this work aims to develop a multi-scale modeling approach employing representative volume elements (RVEs) of optimal size; this is a key issue in asphalt modeling for high-fidelity fracture modeling of heterogeneous asphalt mixtures. To determine the optimal RVE size, a convergence analysis of homogenized elastic properties is conducted using two types of RVEs, one made with polydisperse spherical inclusions, and another made with polydisperse truncated cylindrical inclusions, each aligned with the American Association of State Highway and Transportation Official’s maximum density gradation curve for a 12.5 mm Nominal Maximum Aggregate Size (NMAS). The minimum RVE lengths for this NMAS were found to be in the range of 32–34 mm. After the optimal RVE size for each inclusion shape is obtained, computational models of heterogeneous Indirect Tensile Asphalt Cracking Test samples are then generated. These models include the components of viscoelastic mastic, linear elastic aggregates, and cohesive zone modeling to simulate the rate-dependent failure evolution from micro- to macro-cracking. Examination of load-displacement responses at multiple loading rates shows that both heterogeneous models replicate experimentally measured data satisfactorily. Through micro- and macro-level analyses, this study enhances our understanding of the composition-performance relationships in asphalt pavement materials. The procedure proposed in this study allows us to identify the optimal RVE sizes that preserve computational efficiency without significantly compromising their ability to capture the asphalt material behavior under specific operational conditions.

## 1. Introduction

Asphalt is a complex composite material, the performance of which depends strongly on the morphology of its microstructure. Considering heterogenous material properties at the micro level allows for the creation of multi-scale modeling approaches to better evaluate the composite behavior and overall pavement response as compared to traditional modeling with fully homogenized material properties. By accounting for real microstructural characteristics, such as constituent phases, sizes, shapes, orientations, and local properties, multi-scale modeling has the potential to provide more accurate predictions of mixture performance properties that emerge from these microstructural factors. Moreover, it is particularly useful for modeling pavements, which requires the simultaneous investigation of meter-scale domains along with mm-scale responses i.e., micro-fracture, coalesced cracks, dislocations, etc.

Multi-scale modeling concepts have been applied to asphalt materials and pavements [1,2,3]. These approaches can be divided into two types. In the first type, the constitutive behavior of an infinitesimal material point at the macro-scale is described by the homogenized response of a representative sample of the heterogeneous material structure at the micro-scale [4,5,6]. An important assumption of this approach is the scale separation, which is obtained if the structural dimensions are significantly larger than the dimensions of the material inhomogeneities [7,8,9]. To achieve this, a representative volume element (RVE) is defined, such that it statistically represents the entire composite. This RVE should contain sufficient inclusions to ensure that the overall moduli are effectively independent of the surface values of traction and displacement [10,11,12]. Thus, the RVE should be large enough when compared to the characteristic dimensions of the microstructure (e.g., particle sizes) but small enough when compared to the entire body. In the second type, the assumption of scale separation is not required. This can be addressed using multi-scale modeling, which iteratively solves the micro- and macro-problems, informing one another through a feedback loop [13,14].

Multi-scale modeling of granular composite materials, such as asphalt pavements, offers an avenue to gain a much-needed fundamental understanding of their behavior at different length scales. This understanding is pivotal for optimizing pavement design, assessing performance, and defining maintenance strategies, as shown in a recent review by Wang W. and Wang L. [15]. The scope of the review was focused on developing a multi-scale modeling framework involving the use of RVEs for micro-scale characterization to represent various asphalt pavement constituents.

The concept of RVE was developed in the field of materials science in the mid-20th century, with important early contributions by Hershey [16] on the elasticity of anisotropic cubic crystals. While Hershey did not address asphalt materials specifically, the principal approach of RVE is becoming popular in various material fields, including those dealing with asphalt pavement, to study distribution of stresses and other mechanical properties (observed macroscopically) in microstructural behaviors [16,17]. Building upon these works, more recent studies conducted micromechanical analyses on RVEs and derived Young’s modulus, revealing the potential of utilizing crystal properties to estimate asphalt behavior [18,19,20].

Many studies have investigated the microstructural behavior of asphalt materials to determine the appropriate size of the RVE. These studies used various experimental and numerical techniques to study asphalt materials. For instance, by using geometrical analysis and FE simulations, a study by Kim et al. [21] verified that a size of 50 mm is effective as an RVE for dense-graded Superpave mixtures, but much larger scales are found to be required in the case of stone matrix asphalt. A further study by Kim et al. [22] employed a purely statistical-numerical approach based on volume fractions (not grain size) for multigrain features like aggregate area fraction, gradation, orientation, and spatial distribution, and identified RVE sizes of approximately 60 mm. Additionally, Kanit et al. [23] and Pelissou et al. [24] addressed the representative size of the RVE and the uncertainty in estimating effective properties. They also suggested a statistical theory to calculate the minimum RVE size and they found that an RVE of 20 mm × 20 mm × 20 mm was able to represent asphalt mixture behavior.

With values of 50 mm for Superpave and 60 mm RVE sizes for stone matrix asphalt mixtures, several studies use experimental testing in combination with numerical approaches, such as FEM or digital image correlation (DIC), to estimate the suitable RVE size [25,26,27,28]. Further research by Marasteanu et al., Liu et al., Szydłowski et al., Fadil et al., and Li et al. used a number of modeling approaches, such as computational microstructural modeling and fracture simulations, to refine these estimates [29,30,31,32,33,34].

Despite many improvements in this field, multi-scale modeling of asphalt pavement and its integration with experimental observations still has major research gaps. For instance, there is no standard RVE size estimation technique that could be used for all the different types of asphalt mixtures. The current literature has provided varying recommendations, suggesting a lack of consensus and the need for more comprehensive studies to establish uniform guidelines. Furthermore, multi-scale modeling provided an attractive solution in simulating asphalt behavior, but efforts that integrate these models with real field data are limited. Additionally, it imposes considerable challenges for dealing with computational complexity. Research into more efficient computational algorithms and the use of advanced computational resources is essential to overcome these problems. Finally, multi-scale modeling has not yet been adequately employed in advancing sustainable pavement design. Understanding how these models can facilitate a greater incorporation of recycled materials and other sustainable practices during pavement design could offer significant environmental benefits.

**Figure 1:**
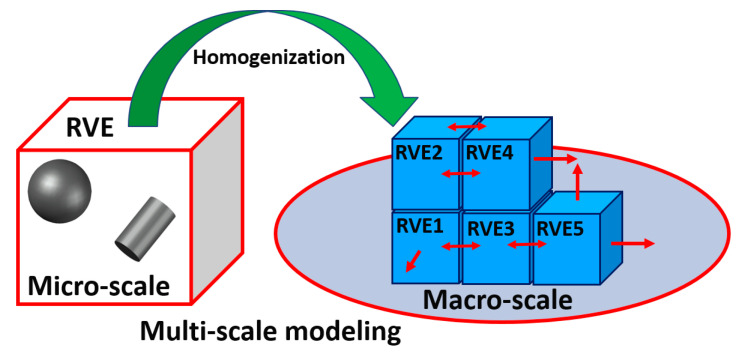
Scheme of multi-scale modeling through RVE based approach.

To address the growing challenges in asphalt pavement design, this study focuses on developing a multi-scale modeling approach to predict damage evolution in heterogeneous asphaltic mixtures. The main objective of this study is to further develop the understanding of how micro-structural features such as particle distribution and binder characteristics control mechanical behavior under realistic loading conditions, as shown in Figure 1.

Building on the literature findings, this study proposes a multi-scale modelling framework for asphalt pavements using RVEs and particle size distributions based on the American Association of State Highway and Transportation Officials (AASHTO) gradation chart. The proposed framework uses RVEs of optimal sizes to create a full-scale IDEAL-CT model that considers viscoelastic mastic behavior and fracture evolution via cohesive zone modeling, for evaluating various asphalt pavement behaviors and mechanical properties under varying loading rates.

One key contribution of this study is the introduction of an approach to determine the RVE size for heterogeneous asphalt mixtures by considering different inclusion shapes (spherical and cylindrical). Our results indicate that the optimal RVE size depends on the inclusion shape, which has not been extensively explored in other studies [4,35,36,37,38,39,40,41,42]. This highlights the increase in sensitivity of microstructural variability, such as inclusion shape, in the modeling of asphalt mixture behavior. A second contribution of this study is the creation of an RVE-based modeling framework capable of reproducing the mechanical behavior of heterogeneous asphalt mixtures under various loading rates. The predictive ability of the proposed framework is demonstrated by comparisons with experimental data obtained from full-scale IDEAL-CT specimens. This represents a critical advance in multiscale modeling of heterogeneous asphalt mixtures as it validates the use of RVEs for evaluating their rate-dependent fracture behavior, a challenge hardly addressed using traditional homogenized models. This work is, however, limited to the scope of determining the optimal RVE sizes based on fixed values for the Young’s modulus of the aggregates and mastic phases. Consequently, the applicability of these optimal RVE sizes may be constrained for different types of aggregates and mastic, or at different temperatures. The latter is an important issue as the apparent elastic properties of the mastic are highly temperature dependent. Further research will aim to address this limitation by exploring the impact of variations in material properties and environmental conditions on the optimal RVE sizes.

## 2. Background

Asphalt pavement consists of aggregate particles coated with a bituminous binder mastic. With such a complex microstructure, homogenization techniques based on RVEs have become an interesting and valuable modeling approach to determine and evaluate the effective properties of asphalt mixtures. In recent years, most of the research studies were focused on the viscoelastic properties of asphalt mixtures, fracture mechanics, and damage evolution models. Some studies took into consideration the effects of binder content, aggregate gradation, and temperature. However, most of the proposed models in past studies lacked detailed simulations at the microstructural level, and such modeling will be in the focus of the present study. Analytically, early models included the rule of mixtures and its variations, including the modified rule of mixtures, along with Halpin Tsai equations for the foundation of estimating elastic properties of composite materials [43,44,45,46]. However, such classical laminate theories experience accuracy limitations, especially when attempting to predict out-of-plane properties. Although the classical theories do provide useful numerical results, they ignore constituent interactions.

Models like the Chamis model, elasticity approach model, self-consistent model, and Mori–Tanaka model are noted for their better prediction capability [39,47,48,49]. The Chamis model has been established as one of the best micromechanical models for determining all five independent elastic properties. While using a similar formulation of the rule of mixtures to estimate Young’s modulus $E_{11}$, which represents the Young’s modulus in the 1-direction along the primary material axis and indicating the material’s stiffness in that direction, and Poisson’s ratio $\nu_{12}$, which describes the ratio of the transverse strain to axial strain when stress is applied in the 1-direction, this model uses different methods for predicting other elastic moduli such as $E_{22}$, which is the Young’s modulus in the 2-direction transverse to the primary axis, representing the stiffness perpendicular to the 1-direction, and $G_{12}$ and $G_{23}$, which represnt shear modulus in the 1–2 plane and 2–3 plane, respectively, representing the material’s resistance to shear deformation between the 1, 2, and 3 directions. The Mori–Tanaka model is a self-consistent method that assumes that each inclusion is embedded in an equivalent particle with the average strain of the composite. This method yields closed-form analytical solutions for computing the five independent elastic moduli of a composite with spherical- or ellipsoidal-shaped inclusions. However, the use of numerical procedures is needed for the composite elastic constants with non-spherical inclusions.

With advancements in computational power, the finite element method (FEM) has become one of the most powerful numerical modeling tools used to model RVEs. FEM facilitates the study of damage mechanisms in composites and complex microstructures [50]. However, generating accurate RVE models is difficult because correctly representing the fine microstructural details of heterogeneous materials requires large databases and computational times. This involves modeling the inter-phase and phase-constituent interactions within the composite (i.e., statistical representativeness, multi-scale material response, etc.). In addition, the computational cost of high-resolution simulations and the challenge to validate models using exactly observed experimental data further complicates this process. While numerous algorithms have been proposed to generate particulate RVE models, these are limited primarily to simple inclusion shapes and low volume fractions [51,52,53]. Computational tools, such as ANSYS Material Designer [54], could be a possible solution to these problems and could facilitate the generation of RVEs tailored for heterogeneous asphalt mixtures. In this study, ANSYS Material Designer is utilized to create a variety of RVEs for homogenized elastic property calculation and for identifying those optimal RVEs that can accurately model heterogeneous asphalt mixtures.

## 3. RVE Modeling and Selection

The development of accurate multi-scale models for asphalt pavement modeling requires an appropriate selection of RVE size while considering adequate aggregate (inclusion) shapes and distribution. In the remainder of this section, we discuss our approach to determining the optimal size of the RVE while considering various types of aggregate shapes with size distributions adhering to AASHTO gradation charts. As will be discussed in detail later, the RVE geometry, as well as the effective elastic properties of the asphalt mixture, will be obtained using ANSYS Material Designer.

### 3.1. RVE Modeling in ANSYS

Two crucial components for accurately predicting the mechanical behavior of asphalt materials within a multi-scale modeling framework are the determination of effective mechanical properties and the selection of a suitable RVE [28,55,56,57].

ANSYS (2022 R2) commercial finite element software was used in this study for modeling RVEs and for simulating fracture behavior in asphalt mixtures. To find the effective mechanical properties of the RVEs, this study employs the ANSYS Material Designer tool. This tool is employed because it simplifies the creation of many types of RVE models and automates the calculation of effective mechanical properties based on aggregate size, distribution, and the material properties of each phase in the RVE. These effective properties could be directly used in macro-scale simulations, or the actual RVEs could be used in heterogeneous modeling. It not only offers several predefined RVE templates, but also allows modeling custom-made RVEs to suit specific needs in an adaptive and efficient way.

While ANSYS is typically used for the macro-mechanical analysis of structures, our work further extends its application by incorporating microstructural features into the RVEs and cohesive zone models (CZM) for modeling of laboratory-scale fracture tests (see Section 4). This provides an approach to not only capture the microscale interactions of aggregates and mastic but also the macroscale performance of asphalt mixture. The use of ANSYS also allowed us to model the viscoelastic behavior of the mastic phase, the most important feature of asphalt mixtures, without requiring additional software development to handle viscoelasticity. Furthermore, certain features of ANSYS, such as the implementation of the exponential cohesive zone model, allowed us to run more realistic fracture simulations. This cohesive law is one of the most widely used laws in modeling fractures in asphalt and other quasi-brittle materials such as concrete, and proved effective in capturing the crack propagation behavior in our asphalt mixture samples [54,58,59,60].

As discussed later, the numerical modeling of typical fracture tests, such as the IDEAL-CT (Indirect Tensile Asphalt Cracking Test), which is widely used within the asphalt pavement community, is performed based on RVEs of optimal sizes obtained in this section. Although ANSYS provides tools for RVE-based modeling, its limitations in full-scale pavement modeling must be acknowledged. For instance, a fully heterogeneous model of an entire pavement section would require the explicit modeling of thousands or even millions of aggregate particles, which is computationally prohibitive. Instead, this study focuses on determining optimal RVE sizes and their applications in laboratory-scale fracture tests. Although out of the scope of the present study, results from such tests could guide the development of equivalent homogeneous fracture models for future full-scale applications, eliminating the need for explicit aggregate modeling.

**Figure 2:**
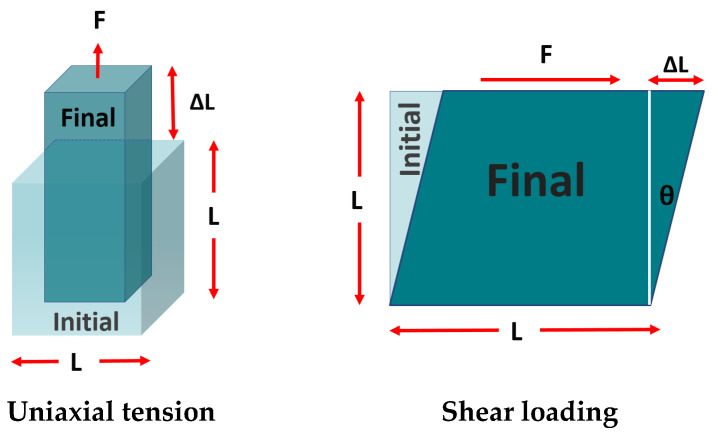
Mechanical characterization of periodic unit cell: load in extension-compression (**left**) and loading in shear (**right**).

ANSYS Material Designer determines the effective material properties of the RVE via homogenization. To do so, the software performs six finite element analyses: tensile tests in the X, Y, and Z directions and shear tests in the XY, YZ, and ZX planes. Figure 2 depicts two representative load cases used to obtain the effective mechanical properties in ANSYS Material Designer. For each test case, a macroscopic unit strain is applied, and the corresponding reaction forces on the boundary faces of the RVE are computed. For each of these analyses, the software imposes periodic boundary conditions on all faces of the RVE. Periodic boundary conditions are typically employed in homogenization approaches to simulate infinite media and mitigate boundary effects [61,62,63]. The material stiffness matrix is then constructed using these reaction forces, from which engineering constants can be derived. The material stiffness matrix D for an orthotropic material is expressed by the below matrix, in which *σ*_*x*_, *σ*_*y*_, and *σ*_*z*_ are the normal stresses in the x, y, and z axes, respectively, and *σ*_*xy*_, *σ*_*yz*_, and *σ*_*zx*_ the shear stresses in the respective planes. The terms *ε*_*x*_, *ε*_*y*_, and *ε*_*z*_ are the normal strains, while *γ*_*xy*_, *γ*_*yz*_, and *γ*_*zx*_ are the respective shear strains. This equation is obtained from the principles of elasticity theory, applying to orthotropic materials, and it describes how the stress components relate to the applied strain components under various loadings [64,65].

$$\left[\begin{array}{c} \sigma_{x}\\ \sigma_{y}\\ \sigma_{z}\\ \sigma_{xy}\\ \sigma_{yz}\\ \sigma_{zx} \end{array}\right]=\left[\begin{array}{cccccc} D_{11} & D_{12} & D_{13} & 0 & 0 & 0\\ D_{21} & D_{22} & D_{23} & 0 & 0 & 0\\ D_{31} & D_{32} & D_{33} & 0 & 0 & 0\\ 0 & 0 & 0 & D_{44} & 0 & 0\\ 0 & 0 & 0 & 0 & D_{55} & 0\\ 0 & 0 & 0 & 0 & 0 & D_{66} \end{array}\right]\;\;\left[\begin{array}{c} \varepsilon_{x}\\ \varepsilon_{y}\\ \varepsilon_{z}\\ {\;\gamma }_{xy}\\ {\;\gamma }_{yz}\\ {\;\gamma }_{zx} \end{array}\right]\;\;$$

Furthermore, the model setup includes full details of the displacement fields at the RVE boundaries to ensure that rigid body motions are restricted, and the periodicity requirements are met. These specifications are critical as they prevent non-physical deformations and maintain the integrity of the homogenization process, thereby providing a more reliable and accurate prediction of the material properties in practical engineering applications [54,55,59,60,66].

### 3.2. RVE Types

**Figure 3:**
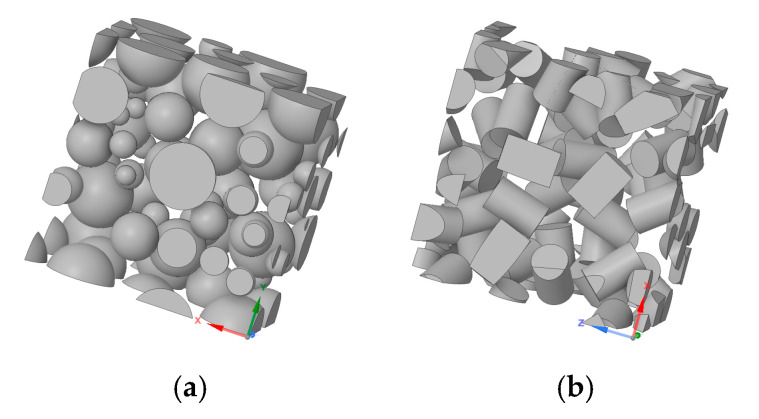
Types of inclusions considered in this study: (**a**) Polydisperse spherical particles and (**b**) truncated cylindrical particles.

As shown in Figure 3, this study considers two types of RVEs. The first considers polydisperse spherical inclusions (Figure 3a), whereas the second considers polydisperse truncated cylindrical inclusions (Figure 3b). The first RVE was considered to capture the effect of different aggregate sizes within the matrix, whereas the second was considered to account for specific material features such as the angularity of aggregate particles. The evaluation of these two types of RVE provided valuable insights concerning the influence of aggregate shape on effective elastic mechanical properties on asphalt pavement materials. 

ANSYS Material Designer requires the user to specify the elastic properties of particles and matrix to obtain the effective mechanical properties of the composite. While this work focuses primarily on the mechanical behavior under the specific loading conditions mentioned, future research will incorporate temperature-dependent properties to simulate asphalt performance in varying thermal conditions. For instance, properties such as thermal expansion coefficient, heat conductivity, and temperature-dependent elastic moduli for the different material phases could be included to account for thermal effects in the RVE model. A model incorporating these thermal properties would allow for analyzing the influence of temperature variations on the optimal RVE size needed for modeling heterogeneous asphalt mixtures. For the purposes of this work, the material properties for both particles (aggregates) and matrix (mastic) are adopted from the literature and are shown in Table 1.

Material properties for the aggregates were selected based on typical values of Young’s modulus (40.5 GPa), and Poisson’s ratio (0.2). For limestone aggregates commonly used in asphalt mixtures, the modulus might vary between 33 and 48 GPa depending on the source material and its composition. In previous studies, the aggregate modulus for asphalt concrete mixtures has been assumed to be approximately 40 GPa. This value agrees well with our chosen value of 40.5 GPa, selected to maintain consistency with the standard industrial practices during the modeling of the elastic behavior of the aggregates in asphalt [67,68,69,70].

This work does not aim to perform a sensitivity analysis of the variation on the influence of Young’s modulus and Poisson’s ratio of aggregates on the optimum size of RVEs. Instead, the effort is focused on using typical values of elastic properties for the different material phases (i.e., aggregates and mastic) and investigating the effect of aggregate shape on mechanical behavior when both aggregate shapes meet the target gradation curves commonly used for asphalt pavements design. The selected elastic properties values have, therefore, been chosen under realistic conditions so that the microstructural interactions of the asphalt mixture can be effectively modeled and analyzed.

The predefined parameters for generating the RVEs containing each of the two inclusion types included volume fraction, initial size, and diameter of particles [55,56]. Next, within the cubical modeled volume, Material Designer randomly places non-overlapping particles to create the RVEs. This process of randomly inserting particles continues until either the volume fraction (i.e., filling level), percolation limit (jamming), or any other primary requirement is satisfied [71,72]. It is important to note that, in all our models, the inclusions were fully contained within the boundaries of the cube. Also, both the matrix and the inclusions were meshed using 10-node tetrahedral elements [58,59,73].

### 3.3. Particle Gradation

**Figure 4:**
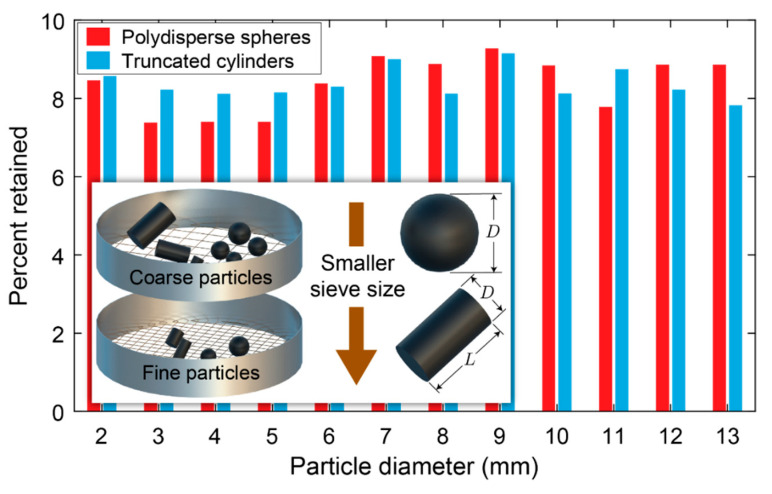
Presentation of each inclusion size in the two selected RVEs.

The size distribution of aggregates plays a crucial role in the performance and engineering properties of asphalt mixtures. Therefore, an appropriate RVE model should accurately reflect the gradations of particles found in real asphalt mixtures. In this context, for the two types of RVEs considered in this study, the percentage of particles present for each sieve size is specified, following a NMAS of 12.5 mm (see Figure 4).

The particle distribution shown in Figure 4 is used to generate gradation curves for each RVE model. These curves were created by first calculating the percentage of particles within the RVE that fall into the 1 mm size range. Particles smaller than 2.36 mm were grouped and treated as homogenized mastic for simplicity, while discrete particles in the range of 2.36–12.5 mm were considered individually [67,74]. Then, by multiplying the percent passing value for each increment by its size, summing these values throughout the range, and dividing by the overall percent passing for the range, the weighted average particle size within each size range was determined.

**Figure 5:**
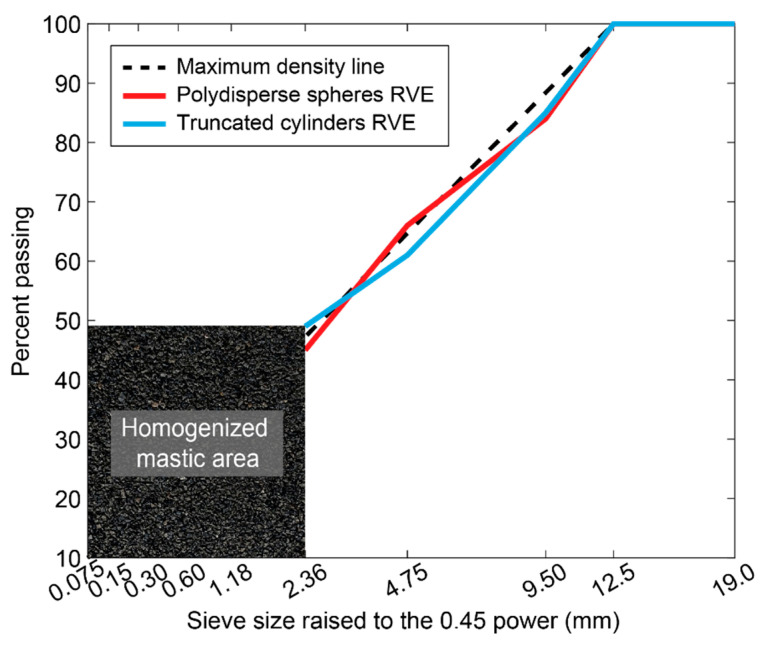
Gradation of RVE particle distribution based on 0.45 power gradation chart.

As depicted in Figure 5, the gradation curve obtained for each RVE is then compared against the 0.45 power maximum density gradation curve per the 12.5 mm size chart specified in AASHTO M323 [75,76]. This 0.45 power curve represents the optimal gradation for dense-graded asphalt mixes. This gradation curve was plotted based on the 0.45 power law, which is generally applied for asphalt mixture designs to achieve optimum packing of aggregates. The law calculates the percentage of aggregate passing through each sieve size so that a well-graded asphalt mixture may be achieved. The X-axis of the plot shows the particle sizes in mm, while the y-axis shows the percentage passing through the respective sieves. The plot has included both the RVE gradation curve and the target 0.45 power gradation curve for comparison. The gradation curve showed a good agreement with the 0.45 power target curve line across all particle sizes for polydisperse spherical inclusion RVEs. Significantly, the truncated cylindrical inclusion RVE was also well-aligned with the 0.45 power gradation curve and had weighted averages matching specified ranges. As the gradation curves of both spherical and cylindrical inclusion RVE agreed well with the target gradation curve, it can be concluded that these two groups of models are appropriate for the representation of real asphalt mixture morphologies. Air voids were not explicitly considered in this study but will be addressed in follow-up research. Air voids are known to reduce the stiffness and strength of asphalt paving materials, and their impact will be an important factor to be considered in subsequent studies [77,78,79].

### 3.4. Optimal RVE Size Selection

To obtain effective elastic properties of heterogeneous asphalt materials using a homogenization approach, the RVE size should be sufficiently large relative to the aggregate size yet small relative to the macroscopic dimensions of the pavement structure. We generated a series of RVE models with side lengths ranging from 24 to 36 mm, in increments of 2 mm, aligning with the NMAS used in our gradation chart. Moreover, for each RVE size, we considered four volume fractions (0.4, 0.5, 0.6, and 0.7).

The optimal RVE size for each type was determined iteratively by increasing their size until the computed effective elastic properties converged, while choosing appropriate aggregate volume fractions that ensure the converged properties align with expected values from the literature. Effective properties for asphalt, typically within 5–7 GPa for E, 1.5–2 GPa for G, and 0.30–0.35 for ν at a standard reference temperature of 25 °C [80,81,82,83], guided our selection. This method allowed us to identify the minimum RVE size necessary to achieve statistically homogeneous and stable material property estimates that are close to the expected values discussed above.

**Figure 6:**
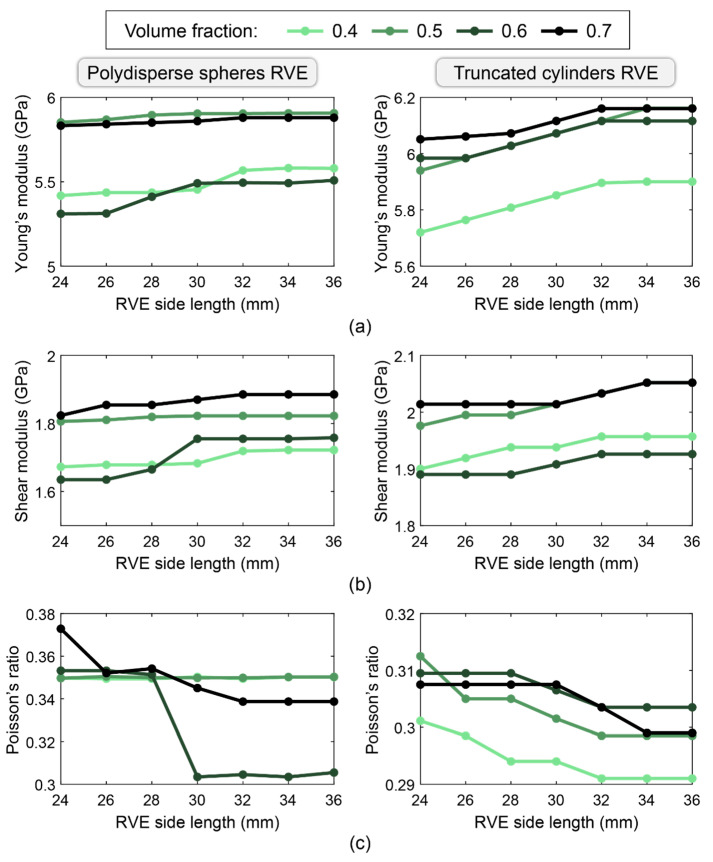
Engineering constants for the two types of RVEs vs. RVE side length for various aggregate volume fractions: (**a**) Young’s modulus, (**b**) Shear modulus, and (**c**) Poisson’s ratio.

Figure 6 shows the convergence analysis of the homogenized elastic properties for spherical and cylindrical RVEs, where the particle distributions are based on the 0.45 power gradation chart. Based on these results and the selection criteria discussed above, the recommended optimal RVE configurations are a 0.5 volume fraction with 32 mm size for the polydisperse spherical RVE and 0.6 volume fraction with 34 mm size for the truncated cylindrical RVE.

For the polydisperse spherical RVE with a 0.5 volume fraction and a 32 mm size, the converged effective properties are E=5.90 GPa (Young’s modulus), G=1.82 GPa (shear modulus), and ν=0.35 (Poisson’s ratio), all of which are within the ranges discussed above. For the truncated cylindrical RVE with 0.6 volume fraction and a 34 mm size, the converged properties are E=6.11 GPa, G=1.93 GPa, and ν=0.30, which are also within the expected ranges. The convergence analysis of homogenized elastic properties shows that the RVE size predictions chosen, 32 mm for spherical inclusions and 34 mm for cylindrical inclusions, can ensure the obtainment of reliable mechanical performances, laying in the expected range given in the literature [25,26,27,28]. The good accuracy of RVE-based models is also ensured by the satisfactory match between the load-displacement curves obtained from output of numerical simulations with those obtained from experimental test results [67] and shown in the following section.

## 4. Full-Scale Models

The optimal RVEs discussed in the previous section were used to create two high-fidelity heterogenous models for an IDEAL-CT. The IDEAL-CT is conducted according to the industry standards, specifically ASTM D8225 [84]. This test evaluates the cracking potential of asphalt mixtures under Mode-I tensile stresses, which simulate the tensile conditions experienced in the field.

The first model considers the optimal RVE with polydisperse spherical inclusions, while the second one considers the optimal RVE with truncated cylindrical inclusions. Unlike the earlier RVE models, which assume linear elastic material properties, these full-scale models incorporate viscoelastic properties for the mastic phase and fracture behavior defined through a cohesive zone model.

Appropriate viscoelastic and cohesive zone properties are defined for the mastic and interfaces, respectively, based on a previous study [67]. Additionally, a mesh convergence analysis is conducted to determine the optimal mesh size. The results from the full-scale models are then compared against experimental results reported in [67]. This validation step involves comparing the load-displacement responses obtained from the models with those obtained experimentally for a range of loading rates. In the remainder of this section, details of the full-scale models and the validated results are provided.

### 4.1. Model Geometry

**Figure 7:**
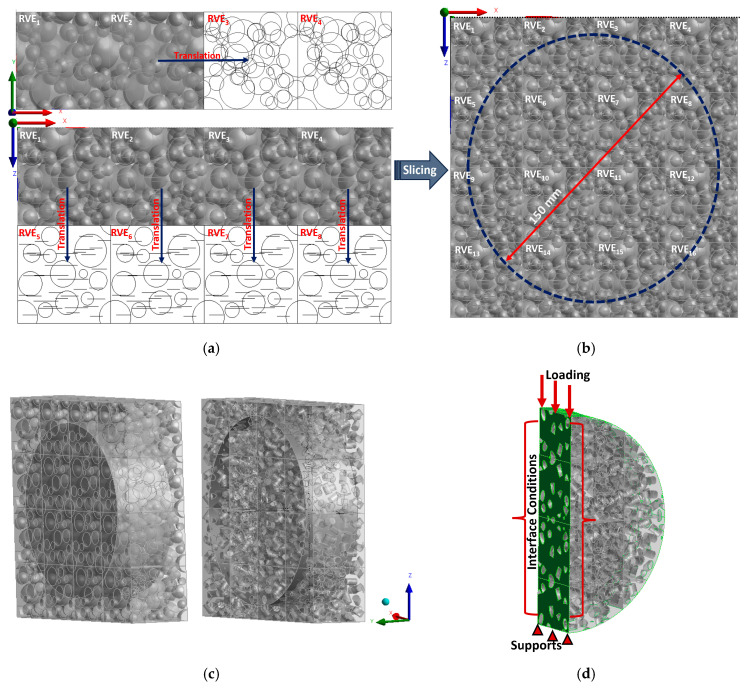
Creation of IDEAL CT full-scale model: (**a**) replicating unit RVE to extend model domain beyond 150 mm × 150 mm × 62 mm, (**b**) slicing cylindrical specimen from extended model domain, (**c**) generated full scale models, and (**d**) applying boundary conditions.

The IDEAL-CT test sample consists of a cylindrical specimen with a diameter of 150 mm and a thickness of 62 mm. Both full-scale models are created using ANSYS workbench. Each model is created by replicating their respective optimal RVE in the *x*, *y*, and *z* directions to fill the volume of the test sample (refer to Figure 7a–c). Given that the optimal RVE size is different when considering polydisperse spherical inclusions or truncated cylindrical inclusions, the number of RVEs occupying each model domain is different for each RVE type. Once the RVEs are replicated, all elements outside the initial cylindrical domain are removed from the model. Finally, for computational efficiency, only half of the IDEAL-CT domains were modeled (refer to Figure 7d).

**Figure 8:**
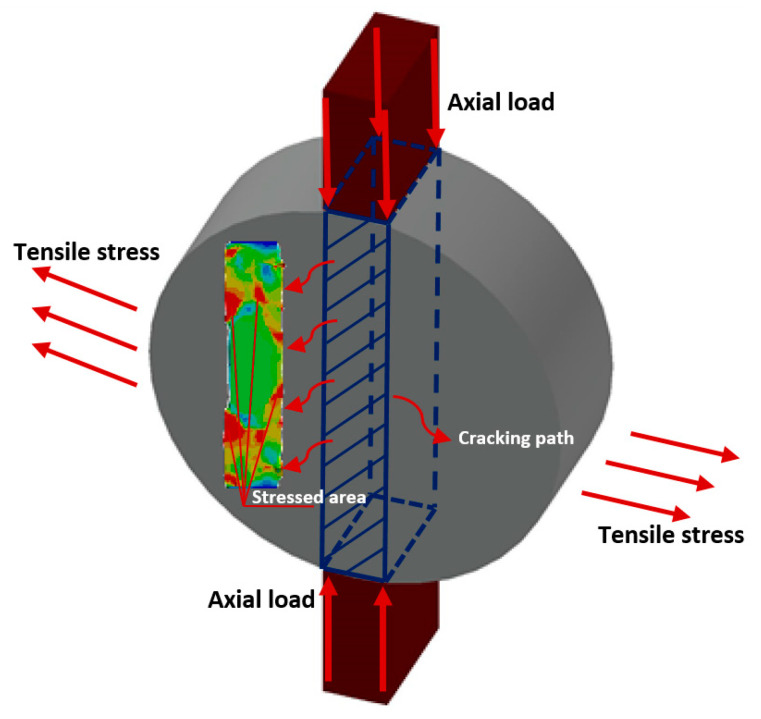
Schematic illustration of a full-scale model depicting the level of damage on the symmetry plane.

Once the domains are generated, cohesive elements are inserted at the mid-plane of the specimens (see Figure 7d) to simulate crack propagation throughout the entire specimen. This was then implemented in the full-scale model validation (see Figure 8) to depict the level of damage on the symmetry plane.

### 4.2. Material Modeling

For the full-scale models, the aggregates are modeled as elastic inclusions with a Young’s modulus of 40,500 MPa and Poisson’s ratio equal to 0.2 [67]. The mastic phase, which is comprised of the asphalt binder, filler content, and fine aggregates, is characterized as a homogeneous viscoelastic material with an 11-term Prony series representation of the relaxation modulus listed in Table 2. Exponential CZMs were used to model the interfaces between aggregate and mastic to simulate fracture propagation at the symmetry plane of the IDEAL-CT specimen.

The values of relaxation modulus and relaxation time used in this study were obtained from data reported by Vasconcelos De Souza et al. [67] in their work on heterogeneous viscoelastic asphaltic mixtures. In their study, to obtain the Prony series terms of the binder’s relaxation modulus, frequency sweep tests were carried out in shear assuming a constant Poisson’s ratio of 0.30. The tests were carried out for a large range of relaxation times over the binder time-dependent viscoelastic behavior. This was used in the modeling of the viscoelastic behavior of asphalt mastic within our numerical simulations so that the relaxation properties exhibited by the binder are correctly captured for analysis.

The cohesive zone approach introduces interface elements that describe the progressive separation of fracturing surfaces through a cohesive traction-separation law. The exponential cohesive zone model (CZM) is advantageous for modeling fracture in asphalt materials exhibiting a gradual softening and damage accumulation ahead of the crack tip [41,67,85]. The exponential CZM defined in ANSYS considers a potential function of the form [86]:

(1) $$\phi (\delta_{n},\;\delta_{t})=e\sigma_{n_{max}}{\bar{\delta }}_{n}\left[1-\left(1+{∆}_{n}\right)e^{{-∆}_{n}}e^{-{∆}_{t}^{2}}\right],$$

where ${\bar{\delta }}_{n}$ is the characteristic normal separation corresponding to the maximum normal traction, $\sigma_{n_{max}}$, and ${∆}_{n}=\delta_{n}/{\bar{\delta }}_{n}$ and ${∆}_{t}=\delta_{t}/{\bar{\delta }}_{t}$ are, respectively, the normalized normal and tangential crack opening displacements, which are expressed in terms of the normal separation, $\delta_{n}$, and tangential separation, $\delta_{t}$. The normal and tangential cohesive tractions are obtained from the potential function in Equation (1). Specifically, the normal traction is given by Equation (2)

(2) $$T_{n}=e\sigma_{n_{max}}{∆}_{n}e^{{-∆}_{n}}e^{-{∆}_{t}^{2}},$$

where $\sigma_{n_{max}}$ is the maximum normal traction, and the shear traction is given by Equation (3)

(3) $$T_{t}=2e\sigma_{t_{max}}\frac{{\bar{\delta }}_{n}}{{\bar{\delta }}_{t}}{∆}_{t}(1+{∆}_{n})e^{{-∆}_{n}}e^{-{∆}_{t}^{2}},$$

where $\sigma_{t_{max}}$ is the maximum tangential traction. The fracture energy in mode I is expressed in Equation (4)

(4) $$\phi_{n}=e\sigma_{n_{max}}{\bar{\delta }}_{n},$$

and the fracture energy in mode II is expressed as presented in Equation (5)

(5) $$\phi_{t}=\sqrt{e/2}\;\sigma_{t_{max}}\bar{\delta_{t}},$$

For both full-scale models, we considered $\sigma_{n_{max}}=\sigma_{t_{max}}=1.32$ MPa and $\phi_{n}=\phi_{t}=960.7$ J/m^2^. These values were selected based on reasonable tensile and normal stress values found in the literature for asphalt mixtures subjected to tensile loading. This fracture energy value reflects the energy required to propagate a crack per unit area in the modeled asphalt mixture [87,88,89].

As outlined in our current study, the next phase of research will involve the application of homogenization techniques to translate the mesoscale behaviors observed in our Representative Volume Element (RVE) models into effective macroscale thermomechanical properties. This transition is crucial for accurately simulating the behavior of asphalt pavements under real-world conditions, where both thermal and mechanical loads play critical roles.

### 4.3. Mesh Convergence Analysis

**Figure 9:**
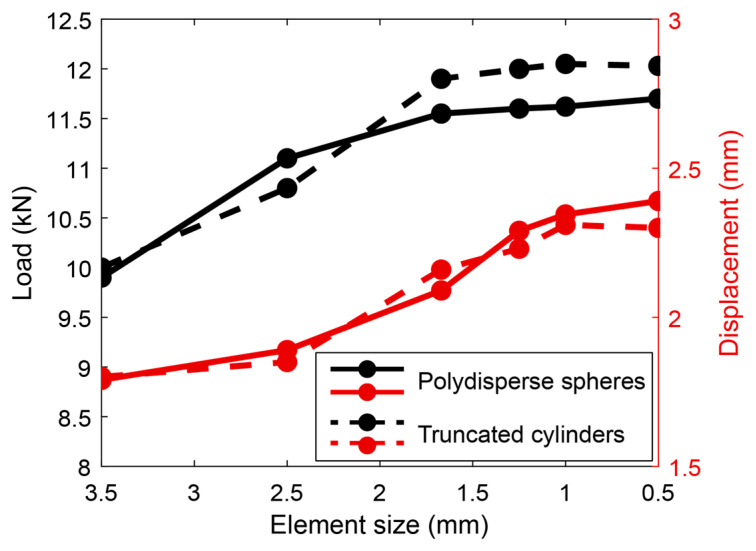
Element size in relation to peak load and maximum displacement.

To ensure the accuracy of our numerical solutions, we conducted a mesh convergence analysis for both full-scale models. As shown in Figure 9, we assessed convergence of the numerical results by monitoring the predicted peak load and maximum displacement as a function of the average element size. This analysis was conducted for both full-scale models by monitoring the predicted peak load and maximum displacement as a function of the element size. For each response parameter, the value from the coarsest mesh (3.5 mm element size) was set as the baseline, and we gradually refined the mesh to assess the variations in the response parameters. Coarse, medium, and fine convergence criteria of 10–15% and 2–3% normalized variation for peak load and maximum displacement from the baseline, respectively, were established based on recommendations in the literature [90,91,92,93]. Initial coarse meshes showed a more than 12% difference in peak load between the 3.5 mm and 2.5 mm elements, reducing below 8% when refined to 1.5 mm elements. Further refinement to 1.25 mm, 1 mm, and 0.5 mm resulted in less than 3% variation, indicating convergence as per the criteria. The optimized mesh size of 1.25 mm was then implemented in the full-scale model validation.

### 4.4. Validation and Experimental Considerations

**Figure 10:**
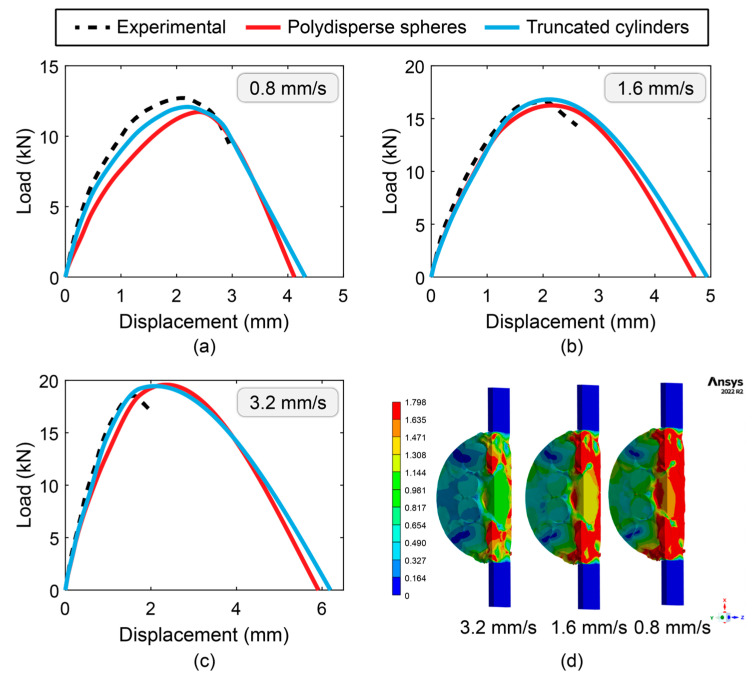
Numerical simulation vs. experiment for load-displacement relation at different loading rates: (**a**) 0.8 mm/s, (**b**) 1.6 mm/s, (**c**) 3.2 mm/s, and (**d**) total deformation (in mm) at the peak load for all loading rates.

This section deals with the validation of the full-scale models presented previously and provides numerical results. These numerical results were validated by comparing them with experimental results provided in Vasconcelos de Souza et al. [67] and Soares et al. [68]. Proprietary testing was carried out in laboratory-controlled conditions on asphalt mixtures with a nominal maximum aggregate size of 12.5 mm at various displacement rates, 0.8 mm/s, 1.6 mm/s, and 3.2 mm/s, recording the corresponding load-displacement curves. These experimental data provided a direct comparison to the numerical simulation results and ensured that the rate-dependent fracture behavior of the asphalt mixture was captured by the model. Figure 10 presents stress and deformation contours extracted from the finite element models. These results provide the areas of high stress and can be used to gain an understanding of how cracks initiate and propagate. As shown by the results in Figure 10a–c, the load-displacement responses predicted by both full-scale models (i.e., the one using polydisperse spherical inclusions and the one using truncated cylindrical inclusion) are in reasonable agreement with the experimental results. In Figure 10d, the contour plots illustrate the total deformation (in mm) at the peak load for each of the displacement rates. The contour plots clearly depict the areas of large deformation, particularly around the midplane of the specimens, which become more pronounced as the displacement rate decreases.

**Figure 11:**
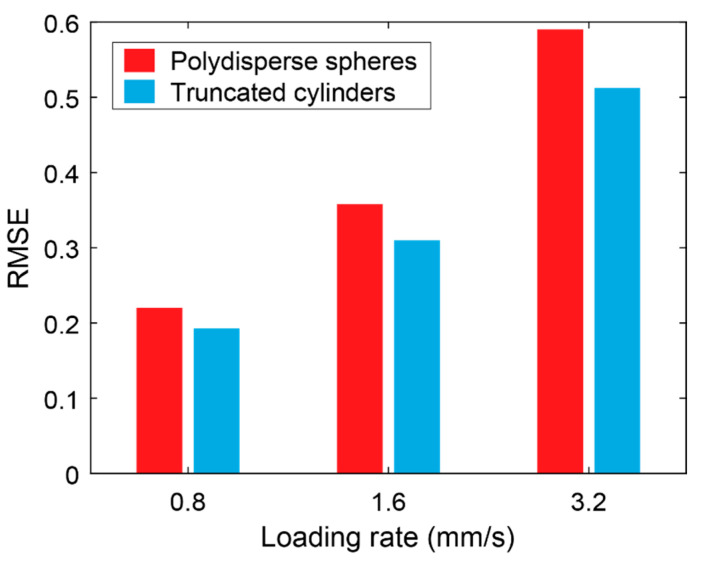
Root mean squared error comparison between the two proposed models vs. the applied loading rates.

In addition to qualitatively comparing the load-displacement curves at different loading rates, we provide a quantitative assessment of the predictive capabilities of each full-scale model. To do so, we computed the root mean squared error (RMSE) for each model and for each loading rate. The computed RMSE values for each model are shown in Figure 11. As shown in the figure, for the spherical inclusions model, the RMSE values generally increased as the loading rate increased, demonstrating the reduced accuracy at higher loading rates. This trend is also observed for the truncated cylinder model, though the RMSEs were consistently lower in these models, indicating an improved fit to the experimental results.

The results discussed above indicate that the full-scale models, built using the optimal RVEs obtained in Section 3, are adequate to predict the behavior of the IDEAL-CT tests under various displacement rates. However, it is important to note that the current models with the assumed RVE sizes may be inadequate to model the behavior of IDEAL-CT tests at different temperatures. This is because the optimal RVE size may be different for different temperatures since the Young’s modulus of the mastic is temperature dependent. This observation points to the need for further studies investigating the optimal RVE size for heterogeneous asphalt mixtures at different temperatures, which can enable the creation of reliable full-scale models for heterogeneous asphalt modeling. Besides the possibility of temperature-dependent RVE sizes, heterogeneous fracture modeling at various temperatures may require more sophisticated cohesive zone models that can capture rate- and temperature-dependent fracture behavior. Our previous work has investigated rate-dependent cohesive-zone models [94,95] and can be used as a basis for future modeling efforts in this area.

## 5. Conclusions

The main objective of this study was to develop a multi-scale modeling framework for characterizing microstructure-performance relationships in asphalt pavement materials. An RVE-based approach was adopted by combining micromechanical homogenization and full-scale modeling. At the micro-scale, optimal RVE sizes were identified through a size-convergence analysis. Convergence analysis was assessed by considering different inclusion shapes and volume fractions. The first type of RVE considered polydisperse spherical inclusions whereas the second type considered truncated cylindrical inclusions. The inclusion size distribution for each RVE type was selected to agree with target gradation curves from AASHTO. The results from the size convergence analysis showed that the minimum RVE size for spherical inclusions was 32 mm, whereas that for truncated cylindrical inclusions, the size was 34 mm. The converged RVE sizes were obtained by analyzing the convergence of the effective elastic properties of the asphalt composite.

Upon determining the optimal sizes of the two types of RVEs, we created two full-scale, heterogeneous models for an IDEAL-CT test. The first model considered polydisperse spherical inclusions whereas the second considered truncated cylindrical inclusions. The heterogeneous models were created by replicating the optimal RVEs in the three special dimensions and performing a Boolean operator whereas elements outside the IDEAL-CT specimen geometry were removed. Unlike the RVE size convergence study, the full-scale models considered material nonlinearities and fracture. The models consisted of aggregates (either spherical or cylindrical), mastic, and interfaces. The aggregates were modeled as linear elastic materials whereas the mastic was modeled by using a viscoelastic material model. To simulate fracture evolution, we inserted cohesive elements at the interface between aggregates and mastic and at the mid-plane of the specimen geometry, where cracks are expected to propagate. The numerical results from the full-scale models were compared against experimental results obtained from the literature, providing a means of validating our multi-scale modeling approach.

While the current model focused on the mechanical behavior of asphalt mixtures without considering variations in chemical composition, future work will explore how changes in binder type, aggregate content, and additives may influence the viscoelastic properties. Similarly, variations in aggregate gradation can impact the load distribution and damage evolution within the material.

The key findings from the study are listed as follows:

1. From the sensitivity analyses, it was concluded that polydisperse spherical and truncated cylindrical RVEs are valid representations of asphalt mixture microstructure at minimum sizes of 32 mm and 34 mm, which fall within the typical range of 20 mm to 60 mm found in the literature.
2. Full-scale, heterogeneous IDEAL-CT finite element models were developed that accurately represented specimen geometry, material phasing, and test characteristics for relevant loading rate effects.
3. Simulations of experimental monotonic fracture tests confirmed the effectiveness of truncating cylindrical inclusion modeling, based on the proposed RVE-based multi-scale approach, to adequately capture the overall behavior, as compared with polydisperse spherical inclusion modeling.
4. The cylindrical inclusion model was found to be slightly more computationally efficient over the spherical inclusion model, which was based on the computed RMSE in terms of predicted vs. modeled load-deflection curves.
5. The proposed multi-scale modeling framework could link microstructure-performance and allow for virtual characterization through full-scale analyses, establishing a foundation for prediction and evaluation of full-scale composite system designs.
6. Potential future work could involve including temperature and moisture effects along with considering the addition of recycled materials as a third phase for better understanding of the effect of varying environmental conditions on durability and fracture behavior of asphalt mixtures.

## Figures and Tables

**Table 1:** Elastic material properties of the homogenized RVE components.

Material	Density (kg/m^3^)	Young’s Modulus (GPa)	Poisson’s Ratio
Aggregate	2300	40.50	0.2
Mastic	1050	2.025	0.3

**Table 2:** Prony series terms for binder relaxation modulus [67].

Term *i*	Relaxation Modulus $E_{i}$ (MPa)	Relaxation Time $\tau_{i}$ (s)
∞	57.36	
1	23.28	1.098 × 10^6^
2	34.56	2.307 × 10^4^
3	64.32	1.056 × 10^3^
4	162.5	1.440 × 10^2^
5	246.0	13
6	6082	1.05
7	4488	0.15
8	5947	0.016
9	6744	0.00029
0	5700	0.000026

## Data Availability

The original contributions presented in the study are included in the article, further inquiries can be directed to the corresponding author.
